# HPV self-sampling and physician-collected sampling for detection of precancerous lesions of the cervix

**DOI:** 10.6026/973206300221597

**Published:** 2026-03-31

**Authors:** Jannatul Ferdous, Jakanta Faika M.S.T, Noor E Ferdous, Farzana Sharmin, Sayeda Fatema Khatun, Monowara Begum, Latifa Akhter, Jawad Khan

**Affiliations:** 1 Department of Gynaecological Oncology, Bangladesh Medical University, Dhaka, Bangladesh; 2 Department of Gynaecological Oncology, National Institute of Cancer Research & Hospital (NICRH), Dhaka, Bangladesh; 3 Department of Biomedical Engineering, Post Masters Independent Researcher, Chart Industries, USA

**Keywords:** HPV testing, HPV self-sampling, clinician sampling, high-risk HPV, physician sampling

## Abstract

Collection of samples plays a pivotal role in detection of precancerous lesion. Hence, comparative study was done on 95 women to assess the performance of self-collected vaginal samples versus physician-collected cervical samples for HPV detection. HPV positivity was similar between self (14.7%) and physician-collected samples (15.8%), with comparable genotype distributions. Physician sampling detected more cervical intraepithelial neoplasia (CIN) lesions and showed higher sensitivity, whereas self-sampling demonstrated better specificity and similar overall accuracy. There was significant difference between both the parameters. Thus, physician-collected cervical sample is better than self-collected vaginal samples.

## Background:

In 2020, the World Health Organization (WHO) reported approximately 604,000 new cases of cervical cancer diagnosed worldwide, with an estimated 342,000 deaths. Notably, 84% of women diagnosed with cervical cancer were from low-resource countries [1]. In Bangladesh, cervical cancer is the second most common cancer in women. In 2020, there were approximately 8,268 new cases of cervical cancer in Bangladesh. The crude incidence rate of cervical cancer in Bangladesh is higher than the global rate but lower than the rate in South Asia [2]. Human papillomavirus (HPV) infection is a major contributor to cervical neoplasia and is detected in 99.7% of cervical carcinomas [3]. Cervical carcinoma is now recognized as the most preventable type of cancer due to the availability of various preventive methods, such as primary prevention through HPV vaccination and secondary prevention via effective screening tests [4, 5- 6]. Risk factors associated with HPV-related cervical cancer include early onset of sexual activity, having multiple sex partners, a history of sexually transmitted diseases, prior vulvar or vaginal squamous intraepithelial neoplasia or cancer, smoking and human immunodeficiency virus (HIV) status [7]. Currently, HPV detection plays a significant role in gynecological practice. It is used in conjunction with cytological examination (cotesting) for primary cervical cancer screening, triage of abnormal cytology results (including ASC-US) and follow-up after treatment of CIN2 and CIN3 lesions, commonly referred to as the "test of cure." Women with abnormal cytology results or positive HPV detection from cervical cancer screening are typically referred for colposcopy. This procedure aims to exclude invasive cervical cancer, identify and treat high-grade lesions before they progress to cancer and determine the appropriate follow-up care. Women with high-risk HPV infections, including HPV16 and HPV18, along with 12 additional high-risk HPV genotypes, face an increased risk of developing cervical cancer.

The American Society for Colposcopy and Cervical Pathology recommends that women aged 25 years and older who have positive HPV tests and abnormal cytology be referred directly for colposcopy [8]. If a high-grade lesion is suspected, a biopsy should be performed. Recent advancements in cervical cancer screening technology have enabled self-collection of specimens (self- collected vaginal samples), which benefits women who do not undergo pelvic examinations or who have limited access to medical facilities. Although current guidelines do not classify self-HPV collection as a standard screening tool, recent studies indicate that cervical cancer screening via self-collection is acceptable and may be as effective as the use of physician-collected samples. This option could be appealing to women who are uncomfortable with pelvic examinations, potentially leading to an increase in the number of women participating in cervical cancer screening. Consequently, this approach may increase patient compliance with ongoing follow-up, reduce the need for hospital visits or pelvic exams and ultimately decrease the incidence of cervical cancer in the future [9, 10, 11, 12, 13-14]. The primary aim of this study was to evaluate the effectiveness of the genital self-sampling human papilloma virus (HPV) test as a screening tool for detecting cervical precancerous lesions. Self-sampling for HPV testing offers a potentially accessible, less invasive alternative to clinician-based sampling and comfort for women. Therefore, it is of interest to compare the detection rates of high-risk HPV infection between self-sampling and clinician- sampling techniques. Ethical clearance and written consent were assured before the study.

## Objective:

To compare the performance of self-collected vaginal specimens with that of physician-collected cervical specimens for the detection of HPV.

## Methodology and Materials:

This comparative cross-sectional analytical study was conducted at the colposcopy clinic of the Department of Gynecological Oncology, Bangabandhu Sheikh Mujib Medical University (BMU), Dhaka, Bangladesh, between December 2022 and January 2024. A total of 95 women aged over 25 years were included in the study, who were selected based on specific inclusion and exclusion criteria for the evaluation of self-sampling versus clinician-collected sampling for the detection of precancerous cervical lesions.

## Inclusion criteria:

[1] Women whose Pap smears were ASCUS (Atypical Squamous Cells of Undetermined Significance) or worse.

[2] VIA (Visual Inspection with Acetic acid) positive women.

[3] Women with unhealthy-looking cervices.

[4] History of postcoital bleeding.

[5] Persistent inflammatory smears.

[6] Women attending the colposcopy clinic of BMU during the study period.

## Exclusion criteria:

[1] Women referred for colposcopy with an HPV-DNA positive test.

[2] Presence of any obvious cervical growth.

[3] Previous procedures on the cervix (*e.g.*, cold coagulation, cryotherapy, conization).

[4] Pregnant women.

[5] Menstruating women.

[6] Hysterectomized women.

After obtaining approval from the Institutional Review Board (IRB) and written informed consent from all participants, detailed information on demographic characteristics, reproductive history and contraceptive history was collected using a structured questionnaire. Participants were instructed on self-collection of vaginal samples via pictorial guides, after which clinician-collected cervical samples were obtained under visual control using Roche kits. Both self-collected and clinician-collected samples were sent to the Department of Virology, BMU, for HPV identification and genotyping via PCR. Colposcopy-guided biopsies were performed from all abnormal areas, or, if no abnormality was observed, a 4-quadrant biopsy from the squamo-columnar junction was obtained. All biopsy specimens were preserved in 10% formalin and sent to the Department of Pathology for histopathological evaluation, which served as the gold standard for determining the sensitivity, specificity, positive predictive value, negative predictive value and accuracy of self-sampling HPV testing compared with physician-collected samples. Data were analyzed using SPSS version 22; mean values were compared using analysis of variance and frequency distributions were analyzed via chi-square or Fisher's exact tests as appropriate. Sensitivity and specificity analyses were performed between the two sampling methods and histopathological findings, with a P-value <0.05 considered statistically significant.

## Results:

A total of 95 women participated in this study, with the majority aged 30-39 years (51.6%), followed by those aged 40-49 years (26.3%); smaller proportions were under 30 years (12.6%), 50-59 years (8.4%) and over 60 years (1.1%) (Table 1 - see PDF). Age-specific HPV DNA detection showed that in self-collected samples, 14 were positive, most frequently in the 30-39 years group (35.7%), followed by 50-59 years (28.6%), 40-49 years (21.4%) and under 30 years (14.3%). In physician-collected samples, 15 were positive, with the highest proportion also in the 30-39 years group (53.3%), followed by 40-49 years (26.6%), under 30 years (13.3%) and 50-59 years (6.6%) (Table 2 - see PDF). Genotype distribution analysis revealed that in physician-collected samples, 89.5% were negative, while HPV 16 and HPV 18 were detected in 7.4% and 1.1% of cases, respectively and other high-risk HPV types in 2.1%. Similarly, in self-collected samples, 90.5% were negative, with HPV 16/18 detected in 7.3%, indicating comparable detection of oncogenic HPV genotypes by both methods. Histopathological evaluation of HPV-positive cases showed that in self-collected samples, CIN I accounted for 14.3%, CIN II for 7.1% and CIN III for 7.1%, whereas in physician-collected samples, CIN I was found in 26.7%, CIN II in 13.3% and CIN III in 6.7% of cases, suggesting a slightly higher diagnostic yield with physician sampling (Table 3 - see PDF). Comparison of self-collected and physician- collected samples with histopathological reports showed that in self-samples, 2.1% were CIN I, 1.1% CIN II and 1.1% CIN III, while in physician samples, 4.2% were CIN I, 2.1% CIN II and 1.1% CIN III (Table 4 - see PDF). Regarding diagnostic performance, physician-collected samples had higher sensitivity for CIN I (85.0% vs. 76.0%) but lower specificity (78.6% vs. 85.7%), while self-samples showed slightly better accuracy (67.4% vs. 63.2%). For CIN II, physician samples had higher sensitivity (84.3%) and accuracy (87.0%), though self-samples had a marginally better PPV (78.9% vs. 75.0%). For CIN III, both methods showed similar sensitivity (53.1% vs. 50.0%), with physician samples achieving higher NPV (98.7% vs. 90.5%) and accuracy (78.9% vs. 75.5%) (Table 5 - see PDF).

## Discussion:

In this study, the estimated detection rates of high-risk HPV (HR-HPV) were compared between clinician-collected cervical specimens and self-collected vaginal specimens. Both techniques detected HPV at comparable rates. Testing for HPV via self-collected samples was reported to be highly acceptable, comfortable and safe [15, 16]. However, the accuracy of new combinations of assays and self-sampling devices has been evaluated in a diagnostic setting and their clinical performance needs further validation [17]. A study conducted in India reported a high prevalence of HPV DNA, with 78.1% of cervical samples and 77.2% of vaginal samples testing positive. The overall agreement between the two sampling methods was 93.9% [18]. In Japan, clinician-collected and self-collected samples showed similarly high positivity rates of 51% and 50%, respectively, with a kappa value of 0.76, indicating strong agreement [19]. Research has also indicated that more oncogenic HPV types are identified in clinician-collected samples than in patient-collected samples, with fair agreement (k = 0.45) [20]. In Ghana, a study reported an overall concordance of HPV detection between self-collected and clinician-collected samples of 94.2%, demonstrating excellent agreement (k = 0.88) [21]. However, the current findings revealed a lower prevalence of oncogenic HPV-16 infection, with 14.7% in self-collected samples and 16.8% in clinician-collected samples. In the Netherlands, HR-HPV prevalence was lower for both sampling methods, at 8.0% for clinician-collected samples and 10.0% for self-collected samples, with a high concordance rate of 96.8% [22]. A separate study in Nigeria reported moderate agreement between self-collected and clinician-collected samples for HPV detection (k = 0.47) [23]. In this study, HR-HPV detection rates were 7.2% for self-collected samples and 6.8% for clinician-collected samples. In Tanzania, HR-HPV prevalence was lower in self-collected samples (13.8%) than in clinician-collected samples (19.0%), although overall agreement was good at 90.5% [24].

In Ethiopia, HR-HPV prevalence was 17.2% in self-collected samples and 15.5% in clinician-collected samples, with moderate agreement (k = 0.58) [25]. Another study reported an HR-HPV prevalence of 14.0% in self-collected samples [26]. Numerous studies have indicated high acceptability rates for self-sampling methods, whether for HPV testing or Pap smears. However, assessing acceptability among different populations is crucial, as cultural attitudes toward pelvic examinations and self-sampling vary across ethnicities and religions [14]. This study reinforces that self-sampling is well accepted among women. In addition to comparable effectiveness to traditional methods, HPV self-sampling outside clinical settings can broaden women's choices for cervical cancer screening and promote earlier detection. In many regions with a high burden of cervical cancer, self-sampling may be the only viable option to improve access and uptake. It is now considered a key component in achieving the WHO's cervical cancer elimination targets. Despite high satisfaction with self-sampling, a considerable percentage of women still prefer clinician-collected sampling for their next test [14]. This preference may reflect the belief that healthcare providers can perform sampling more effectively, as well as traditional beliefs or personal attitudes favoring conventional methods. The diagnostic performance of HPV DNA detection in self- and physician-collected samples demonstrated that physician-collected samples had slightly higher sensitivity for CIN I and CIN II, while self-collected samples showed higher specificity across all CIN grades, with comparable accuracy for CIN I-III. These findings are consistent with Yan *et al.* [27], who reported no significant difference in sensitivity between vaginal self-collected and clinician-collected cervical samples for CIN II+ detection, although vaginal samples had higher specificity. Similarly, Inturrisi *et al.* [17] found that self-collected vaginal specimens performed comparably to clinician-collected samples in detecting CIN III+ lesions. Kamath & Withers [28] review of studies from low and middle-income countries further supports these results, highlighting that self-sampling achieves high sensitivity and specificity, particularly when compared with colposcopy as the gold standard. Overall, this study reinforces that HPV self-sampling is a reliable alternative to clinician-collected sampling for detecting precancerous cervical lesions, providing an effective option to expand screening coverage without compromising diagnostic performance.

## Conclusion:

We show that the efficacy of HPV detection using self-collected samples is nearly comparable to physician-collected samples for the diagnosis of cervical intraepithelial neoplasia (CIN). Therefore, HPV self-sampling is a highly satisfactory method and provides an additional option to enhance cervical cancer screening coverage.
